# Chemical and Structural Characterization of *Eucalyptus* sp. Wood Residue–Poly(vinyl acetate) Composites

**DOI:** 10.3390/polym18212652

**Published:** 2026-10-26

**Authors:** Gilson Mendonça de Miranda Júnior, Nathalia Gabriele Franca Dias Polido, Michel Picanço Oliveira, Josinaldo de Oliveira Dias, Edgar Vladimiro Mantilla Carrasco, Rejane Costa Alves

**Affiliations:** 1 Graduate Program in Chemical Engineering, Department of Chemical Engineering, Federal University of Espirito Santo, Rua Alto Universitário, S/N, Alegre 29500-000, ES, Brazil; nathaliagfdias@gmail.com; 2 Department of Forestry and Wood Scinces, Federal University of Espírito Santo, Av. Governador Lindemberg. 316, Jerônimo Monteiro 29500-000, ES, Brazil; michel.oliveira@ufes.br (M.P.O.); josinaldo.dias@ufes.br (J.d.O.D.); rejanealves.ufes@gmail.com (R.C.A.); 3 Department of Architecture and Design, Federal University of Minas Gerais, Rua Paraíba, 697, Savassi, Belo Horizonte 30130-141, MG, Brazil; mantilla.carrasco@gmail.com

**Keywords:** wood–plastic composites, *Eucalyptus* sp., polyvinyl acetate, wood residues, chemical characterization, structural characterization, thermal stability, interfacial adhesion, sustainability

## Abstract

This study investigates the production of wood–plastic composites (WPCs) using *Eucalyptus* sp. residues (RE) and poly(vinyl acetate) (PVAc), aiming to evaluate the chemical, structural, thermal, and morphological characteristics of the resulting composites. The research is motivated by the need to add value to residues from the wood industry in Espírito Santo, Brazil, contributing to more sustainable material utilization. The composites were produced with different proportions of RE (70%, 60%, and 40%) and PVAc (30%, 40%, and 60%) and characterized using thermogravimetric analysis (TGA), Fourier-transform infrared spectroscopy (FTIR), X-ray diffraction (XRD), solid-state nuclear magnetic resonance (NMR), and scanning electron microscopy (SEM). TGA results showed that increasing the PVAc content was associated with a higher initial decomposition temperature and a lower mass loss in the main degradation stage. XRD analysis indicated higher crystallinity in composites with greater RE content, while CP/MAS ^13^C NMR revealed changes in the organization of the cellulose structure with increasing PVAc content, including a relative increase in signals associated with amorphous regions. FTIR analysis showed changes in the spectral profile and chemical environment of the functional groups as the RE/PVAc ratio varied, although the spectra alone did not conclusively establish the formation of stronger hydrogen bonds. SEM analysis revealed changes in the fracture morphology and matrix distribution with increasing PVAc content. Composites containing 70% RE and 30% PVAc exhibited more pronounced interfacial voids and discontinuities, whereas those containing 40% RE and 60% PVAc showed greater matrix coverage and more continuous polymer-rich regions. However, greater matrix coverage does not, by itself, demonstrate higher interfacial adhesion. Overall, the results demonstrate that variations in RE/PVAc ratio and particle size significantly influence the thermal, chemical, structural, and morphological characteristics of the composites, highlighting the potential of *Eucalyptus* sp. residues for the production of value-added wood–plastic composites.

## 1. Introduction

The timber industry, based on planted and managed forests, plays a crucial role in the state of Espírito Santo, representing an important source of economic resources. In 2022, mechanically processed wood production generated a gross value of BRL 26.8 billion, corresponding to 5.8% of the national total [1]. However, the furniture industry production chain generates large amounts of wood residues, which are not always adequately managed, making Brazil one of the major generators of these residues [2].

The main residues generated include sanding dust, sawdust, wood trimmings and offcuts, solvents, and paint booth wastewater [3]. Among these, wood trimmings and offcuts are the most abundant. According to NBR 10004 [4], these residues exhibit characteristics such as combustibility and biodegradability.

In view of environmental concerns, it is essential not only to use sustainable materials but also to ensure the proper disposal and management of residues generated during production. The reuse of these residues for the development of new sustainable materials is the main focus of this study. In this context, furniture industry managers face the challenge of reconciling operational efficiency with the pressing need to adopt practices that minimize environmental impacts [5].

Previous studies have demonstrated the potential of poly(vinyl acetate) (PVAc) for applications involving Eucalyptus wood. Gesso et al. [6] observed satisfactory performance of *Eucalyptus* sp. joints bonded with PVAc, whereas Iejavs et al. [7] evaluated Eucalyptus grandis and found that pressing pressure and pressing time influence joint strength. Although these studies demonstrate the applicability of PVAc to Eucalyptus-based materials, both investigated its use as an adhesive rather than as a matrix in wood–plastic composites produced from particulate wood residues. Therefore, the combination of Eucalyptus residues and PVAc in wood–plastic composites, considering different proportions and particle sizes, represents a relatively unexplored approach.

The literature on wood–plastic composites (WPCs) has focused mainly on the use of thermoplastic polymers as matrices, particularly high-density polyethylene (HDPE), low-density polyethylene (LDPE), polypropylene (PP), poly(vinyl chloride) (PVC), polystyrene (PS), and poly(lactic acid) (PLA) [8]. These polymers have been extensively investigated in combination with different lignocellulosic residues due to their processability and the properties achieved in the resulting composites. However, the use of poly(vinyl acetate) (PVAc) as a matrix in WPCs has received less attention, particularly when combined with Eucalyptus residues, indicating an opportunity to investigate this combination.

The chemical and structural characterization of wood–plastic composites is essential for understanding the interactions between the polymer matrix and lignocellulosic residues, as well as for assessing how formulation and processing parameters influence the quality of the final composites [9]. In this context, this study aims to produce and characterize wood–plastic composites with different residue/PVAc ratios and particle sizes, investigating the effects of these variables and their potential interaction on the chemical, thermogravimetric, and morphological characteristics of the resulting composites, thereby contributing to the valorization of wood industry residues and their application in composite production.

## 2. Materials and Methods

### 2.1. Production of the Wood–Plastic Composite

Poly(vinyl acetate) (PVA) supplied as an aqueous emulsion (Eurocryl 5300, Euroamerican do Brasil, Jandira, São Paulo, Brazil), with a Brookfield RVT viscosity of 5000–7000 cP at 25 °C and a total solids content of 45.00–47.00%, was used as the polymer matrix, while untreated *Eucalyptus* sp. waste was used as the lignocellulosic reinforcement. The waste was collected at a sawmill located in Espírito Santo, Brazil.

**Figure 1:**
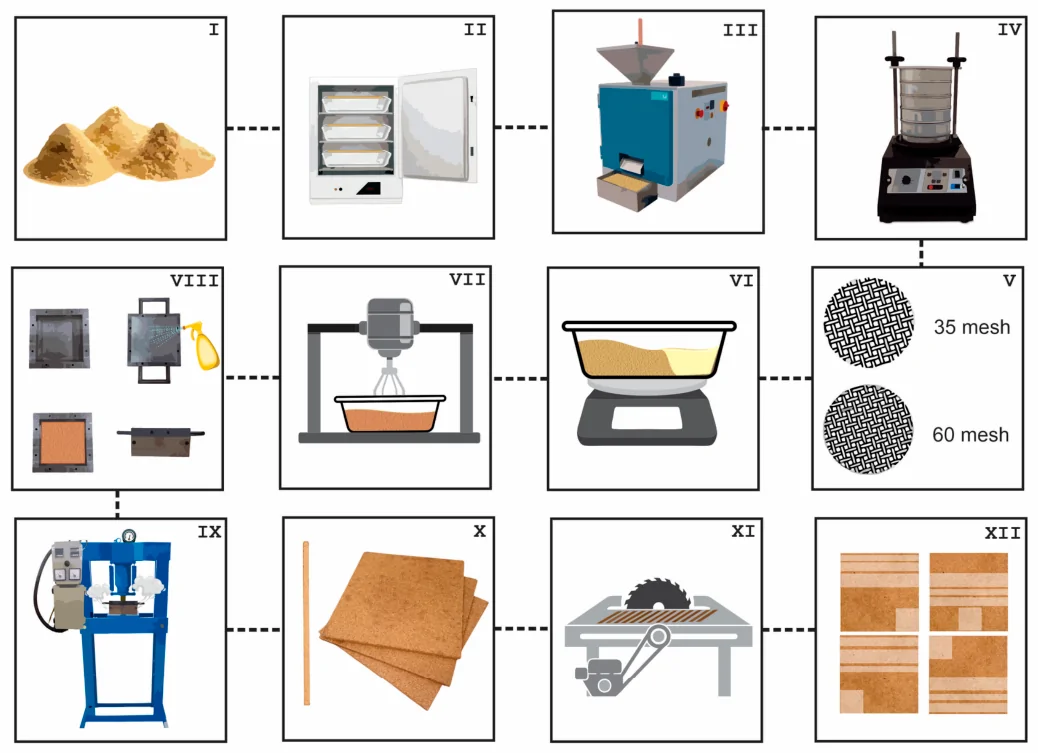
Flowchart of the wood–plastic composite manufacturing process.

The processing procedure is summarized in Figure 1. Initially, the *Eucalyptus* sp. residues were dried in an oven at 100 °C for 24 h to remove moisture (Step II). They were then ground in a domestic-type mechanical mixer (knife mill) (Step III) and sieved through 35- and 60-mesh screens (Steps IV–V). The selected fractions were weighed according to the established formulations (Step VI). For the mixing step (Step VII), the *Eucalyptus* sp. residues were first placed in a domestic-type mixer, and the poly(vinyl acetate) (PVAc) emulsion was subsequently added without prior dilution. The mixture was processed at the maximum available speed of the mixer for 5 min. The batch size varied according to the formulation: Treatments 1 and 4 contained 147 g of *Eucalyptus* sp. residue and 63 g of PVAc; Treatments 2 and 5 contained 126 g of *Eucalyptus* sp. residue and 84 g of PVAc; and Treatments 3 and 6 contained 84 g of *Eucalyptus* sp. residue and 126 g of PVAc. Homogeneity was assessed visually, considering the mixing step complete when the PVAc emulsion had sufficiently wetted the entire amount of wood residue, with no visibly dry portions remaining.

For molding, the mixtures were placed in steel molds previously coated with a release agent (Step VIII) and hot-pressed in a hydraulic press (Bovenau, model P30000, 30-ton capacity, Rio do Sul, Santa Catarina, Brazil) at 150 °C. The pressing cycle included an initial stage at 8–14 metric tons, performed for a minimum of 12 cycles and continued until no further visible vapor was released, indicating the completion of air and moisture removal. This was followed by final consolidation at 20 metric tons for 5 min (Step IX). Square plates with dimensions of 170 × 170 × 6 mm and a target density of 650 kg/m^3^ were obtained (Step X).

Three formulations with different residue-to-matrix ratios (70/30, 60/40, and 40/60, m/m) were produced for each particle size (35 and 60 mesh), totaling six treatments. These particle sizes were selected based on the sieving results, as 35 and 60 mesh showed the highest retention of the *Eucalyptus* sp. residue among the evaluated fractions (Table 1).

Finally, the plates were cut into test specimens using a circular saw (Step XI). The test specimens were randomly positioned within each plate to minimize potential positional effects on the results (Step XII).

### 2.2. Chemical and Structural Characterization

For the chemical and structural analyses (XRD, TG/TGA, NMR, and FTIR), powdered samples were prepared from the wood–plastic composites produced in this study. The samples were obtained by grinding the composites after processing and were subsequently used for the respective analyses. The thermal analysis was conducted following the methodological principles described in ASTM E1131 and ISO 11358 [10,11]. X-ray diffraction analysis was performed to characterize the crystalline structure of the materials, while solid-state ^13^C NMR analysis was conducted according to the methodological approach described by Fortunato [12]. FTIR analysis was used to identify the main functional groups present in the wood residue and PVAc matrix and to compare the chemical characteristics of the composites.

#### 2.2.1. Thermogravimetric Analysis (TG/DTG)

Samples of the composites with the highest concentration of *Eucalyptus* sp. waste (70% RE and 30% PVAc) and the lowest concentration of residues (40% RE and 60% PVAc), both with a particle size of 35 mesh, as well as samples of pure *Eucalyptus* sp. and PVAc, with the aim of evaluating the thermal stability of the composites and identifying the stages of thermal decomposition. Thermogravimetric analysis was performed using a Shimadzu DTG-60H instrument (Shimadzu Corporation, Kyoto, Japan). The thermogravimetric curves were recorded over the range of 10 °C to 800 °C, at a constant heating rate of 10 °C/min, under a nitrogen flow of 50 mL/min. After reaching 800 °C, the samples were exposed to an oxygen atmosphere, also at a flow rate of 50 mL/min, to ensure complete combustion of the material at that temperature. For each test, approximately 6 to 15 mg of material was used, placed in open alumina capsules. The derivative thermogravimetric (DTG) curves were used to identify the main stages of thermal decomposition and their corresponding temperature ranges. The onset decomposition temperature (Tonset) was determined using the tangent method, by identifying the intersection between the tangent to the steepest region of the mass-loss curve and the baseline of the TG curve.

#### 2.2.2. Fourier Transform Infrared Spectroscopy (FTIR)

The Fourier transform infrared (FTIR) spectra of the samples were obtained using an Agilent Technologies Cary 630 FTIR spectrometer (Agilent Technologies, Santa Clara, CA, USA). Samples of pure *Eucalyptus* sp., as well as treatments consisting of 70% RE and 30% PVA, 60% RE and 40% PVA, and 40% RE and 60% PVA, were analyzed using the respective mixture of each treatment with a particle size of 35 mesh after grinding. The method used was ATR (Attenuated Total Reflectance), with 16 scans and a resolution of 4 cm^−1^.

#### 2.2.3. X-Ray Diffraction (XRD)

X-ray diffraction (XRD) analyses of the wood–plastic composites were performed in powder diffraction mode using a glass sample holder. The equipment used was the Shimadzu XRD-6000 diffractometer (Shimadzu Corporation, Kyoto, Japan), with Cu-Kα radiation (λ = 1.5418 Å). Measurements were obtained by scanning the diffraction angle (2θ) over the range of 5° to 60°, with 0.02° increments and a scan speed of 2°/min. The instrument operated at 40 kV and 30 mA. The samples analyzed included pure *Eucalyptus* sp. residues, as well as formulations with a higher concentration of eucalyptus residues (70% ER and 30% PVA) and a lower concentration of residues (40% ER and 60% PVA), with the aim of analyzing the characteristic peaks, identifying the degree of crystallinity and amorphism of the material, and evaluating changes in the peaks as a function of the interaction between the *Eucalyptus* sp. residues and the PVA polymer matrix.

#### 2.2.4. Nuclear Magnetic Resonance (NMR)

NMR spectra were obtained using a 400 MHz Varian/Agilent spectrometer (Agilent Technologies, Santa Clara, CA, USA) with a magnetic field of 9.4 T (frequency of 100.52 MHz for ^13^C (carbon-13)). Samples were analyzed using 4 mm zirconia rotors at room temperature, employing the CP/MAS (Cross Polarization/Magic Angle Spinning) technique for ^13^C, with high-power decoupling (SPINAL) for the ^1^H nuclei. The MAS frequency was 10 kHz, with a 3.6 µs π/2 pulse on the ^1^H nuclei. The acquisition time was 20.48 m/s with a 50 kHz spectral window, accumulating 1500 transients. ^13^C CP/MAS NMR spectra were obtained for five samples: RE and Treatments 1, 3, 4, and 6, with a relaxation time (d1) of 100 s and a π/2 pulse of 4.3 µs. All spectra were processed using ACD/NMR Processor Academic Edition, employing exponential apodization and deconvolution with Gaussian and Lorentzian curves.

#### 2.2.5. Scanning Electron Microscopy (SEM)

For the morphological characterization of the composites using scanning electron microscopy (SEM), samples of approximately 1 cm were obtained from the wood–plastic composites produced in this study. The samples were prepared from the composites after processing and were analyzed to characterize the morphology of their surfaces and the distribution of the PVAc matrix over the Eucalyptus sp. residue. The samples were sputter-coated with a gold alloy using a Quorum 150R ES PLUS system (Quorum Technologies Ltd., Laughton, East Sussex, UK). Images were obtained at magnifications of 100× and 1000× to examine the morphological features of the composites.

## 3. Results and Discussion

### 3.1. Thermogravimetric Analysis (TG/DTA)

Thermogravimetric analyses (TGA) were performed on pulverized samples (fine powder) obtained from composites with higher (70% RE and 30% PVA) and lower (40% RE and 60% PVA) concentrations.

**Figure 2:**
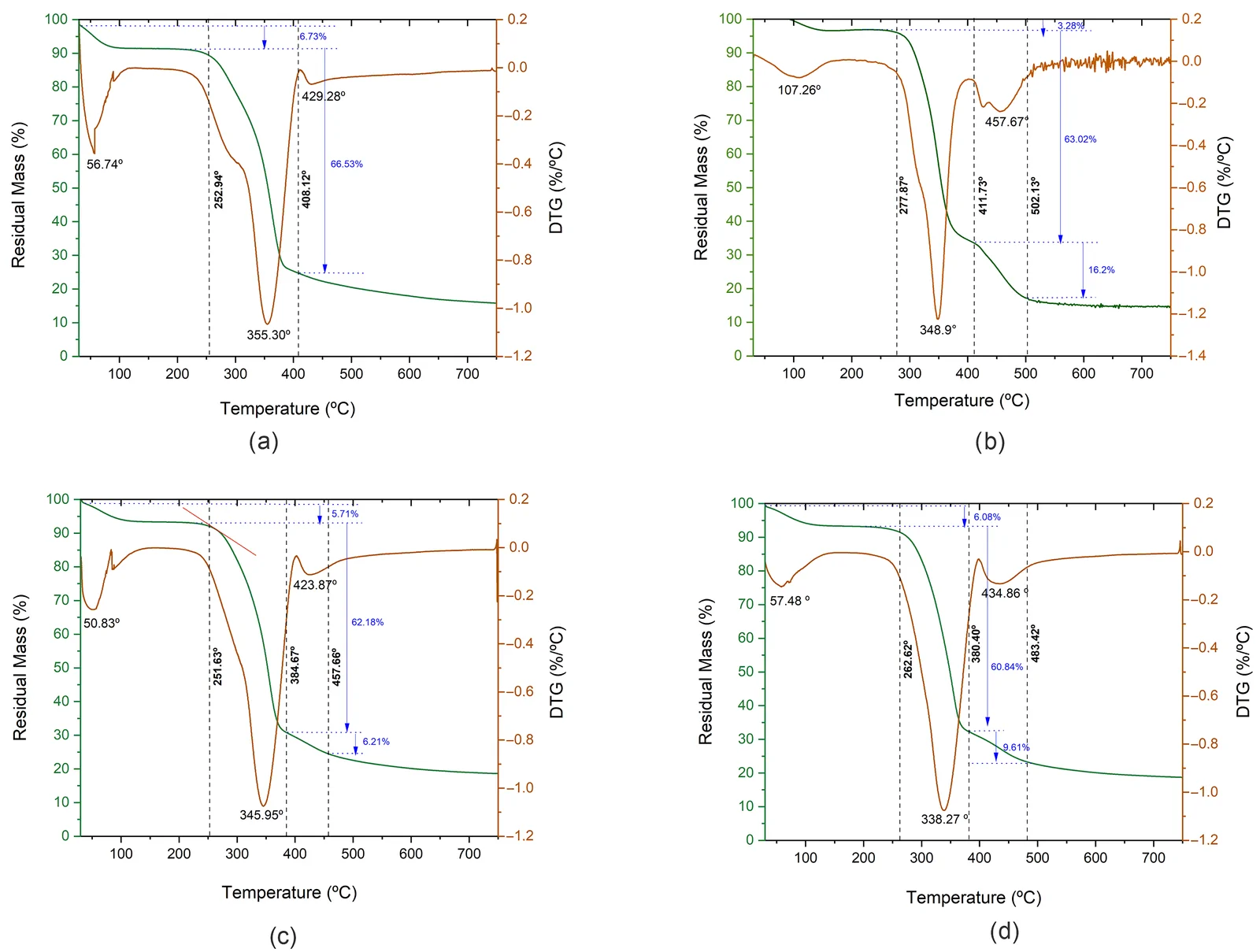
TG and DTG curves: (**a**) pure eucalyptus residue; (**b**) polymer matrix (PVA); (**c**) composite of 70% RE and 30% PVA; (**d**) composite of 40% RE and 60% PVA. Source: the author.

Figure 2 illustrates the thermogravimetric behavior of the treatments, showing only *Eucalyptus* sp. residues (RE), PVA, and the treatments T1 (70% RE and 30% PVA) and T3 (40% RE and 60% PVA). The presence of multiple peaks in the DTG plot indicates the occurrence of different decomposition processes, each associated with a specific component of the eucalyptus biomass, such as moisture (approximately 50–100 °C), extractives (100–200 °C), hemicellulose (200–280 °C), cellulose (280–350 °C), and lignin (350–520 °C), in that order [9,13].

The mass loss of all composites and RE began at approximately 30 °C, as indicated by the DTG curves, and extended to approximately 130 °C. This initial mass loss is attributed primarily to the evaporation of water and volatile organic compounds [14]. The main degradation stage of RE occurred between 252.94 and 408.12 °C, resulting in a mass loss of 66.53% of the sample. This temperature range is associated with the thermal degradation of the main lignocellulosic components, particularly hemicellulose and cellulose [15]. Above 400 °C, the residual mass observed at higher temperatures may be attributed to the formation of charred residues, the presence of inorganic components such as ash, and lignin, which is a structural component of the wood cell wall and exhibits relatively high resistance to thermal decomposition [16].

The thermal behavior of PVAc revealed three main stages of mass loss: an initial loss of 3.28% between 47 °C and 107.26 °C, attributed to the loss of absorbed and bound water; a second stage of 63.02%, beginning at 270.32 °C and peaking at 348.9 °C, associated with the degradation of the polymer chains; and a third stage of 16.21%, reaching the peak of decomposition at 457.67 °C, as reported [17,18]. The differences in the values and final stages of degradation may be related to variations in the composition and characteristics of the polymer, as well as to experimental conditions such as heating rate and atmosphere.

The initial decomposition temperature of the composite containing 70% *Eucalyptus* sp. waste (RE) and 30% PVAc was 251.63 °C, whereas the composite with a higher PVAc concentration (60%) exhibited an initial decomposition temperature of 262.62 °C. Thus, the increase in PVAc content was associated with a shift in the onset decomposition temperature toward higher values. This result indicates that the RE/PVAc composition influences the onset of the main thermal degradation process of the composites.

The main decomposition behavior of the composites was evaluated based on the characteristic decomposition temperatures and mass loss. The composite containing 70% RE and 30% PVAc exhibited a mass loss of 62.18% between 251.8 and 384.67 °C, whereas the composite containing 40% RE and 60% PVAc exhibited a mass loss of 60.84% between 262.62 and 380.40 °C. The composite with the higher PVAc content therefore showed a higher onset decomposition temperature and a lower mass loss during the main degradation stage.

The thermal degradation of lignin and cellulose generally occurs in the temperature range of 300–450 °C [19]. Lignin, being an aromatic polymer with a highly stable structure, retains a greater amount of residual mass at high temperatures, often contributing to char formation. In contrast, cellulose and hemicellulose undergo more pronounced thermal degradation within the temperature range characteristic of the main decomposition of lignocellulosic materials [20,21].

According to Fialho et al. [22], the greatest mass loss of wood occurs in the range of 300–450 °C, associated predominantly with cellulose degradation, which accounts for about 48% of the wood composition. Hemicellulose degradation occurs mainly between 200 and 300 °C, while lignin exhibits a broader degradation range, beginning at lower temperatures and extending above 450 °C. This behavior reflects the different thermal stabilities of the lignocellulosic components.

Overall, the results indicate that the variation in RE/PVAc composition affects the thermal behavior of the composites. The composite containing 40% RE and 60% PVAc exhibited a higher onset decomposition temperature and a slightly lower mass loss in the main degradation stage than the composite containing 70% RE and 30% PVAc. These differences are associated with the relative proportions of the lignocellulosic and polymeric phases and their distinct thermal degradation behaviors [23,24].

### 3.2. Infrared Spectroscopy (FTIR)

**Figure 3:**
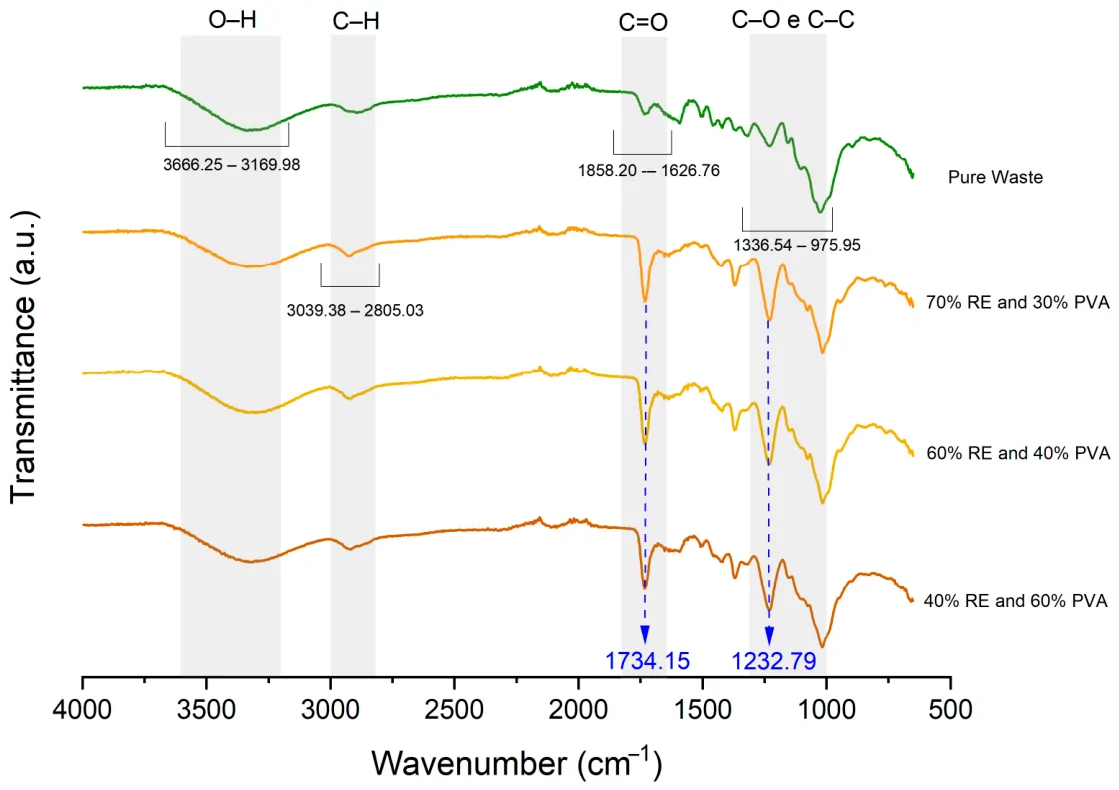
Infrared spectra of the RE, 70% RE and 30% PVA, 60% RE and 40% PVA, and 40% RE and 60% PVA samples. Source: the author.

The FTIR spectra for eucalyptus residue and for the different treatment concentrations (70% RE and 30% PVA, 60% RE and 40% PVA, 40% RE and 60% PVA) are shown in Figure 3.

The FTIR spectra of the composites revealed variations in the intensity and position of characteristic bands, indicating changes in the chemical environment of the functional groups resulting from the different RE/PVAc compositions.

The broad band in the 3666–3170 cm^−1^ region, attributed to the stretching of hydroxyl (–OH) groups in cellulose, hemicellulose, and lignin, showed a decrease in intensity as the PVAc content increased. This behavior, corroborated by Tsioptsias et al. [17], may be associated with changes in the chemical environment of hydroxyl groups and possible intermolecular interactions between the biomass and polymer chains. However, since the RE/PVAc ratio varied among the composite formulations, the decrease in the intensity of the O–H band may also be related to changes in the relative composition of the materials. Although hydrogen bonding between the hydroxyl groups of the biomass and the polymer chains is chemically plausible, FTIR analysis alone is not sufficient to conclusively confirm the formation or increase in the number of these interactions. Therefore, the observed changes should not be interpreted as evidence of new covalent bonds or significant structural modification.

At the same time, an increase in the intensity of the aliphatic C–H vibrations (3039–2805 cm^−1^) was observed. As discussed by Aziz et al. [13], this increase reflects the contribution of methylene groups (–CH_2_–) from the polymer chain, indicating the contribution of the polymer phase to the composition of the material. The band at 1734 cm^−1^, associated with carbonyl (C=O) groups, was more pronounced with the addition of PVAc. This band may be related to carbonyl-containing groups present in the lignocellulosic components, particularly hemicellulose, as well as to the acetate groups of PVAc. Therefore, its increased intensity should be interpreted considering the contributions of both phases and the variation in the RE/PVAc ratio, rather than as direct evidence of the formation of new carbonyl structures or chemical bonds.

In the “fingerprint” region (1336–975 cm^−1^), associated with C–O and C–C stretching vibrations of polysaccharides and ether bonds of lignin [25,26], an intensification of the signals was observed, especially at 1232 cm^−1^. This behavior may be associated with the overlap of vibrational modes from the polymer and lignocellulosic components of Eucalyptus.

Thermal analysis (TG) provides complementary information on the thermal behavior of the materials, particularly regarding their mass loss and thermal degradation. Overall, the FTIR results indicate changes in the spectral profile and chemical environment of the functional groups as a function of the composite composition. However, the observed changes do not provide sufficient evidence to confirm the formation of new covalent bonds or stronger hydrogen bonding interactions.

### 3.3. X-Ray Diffraction (XRD)

**Figure 4:**
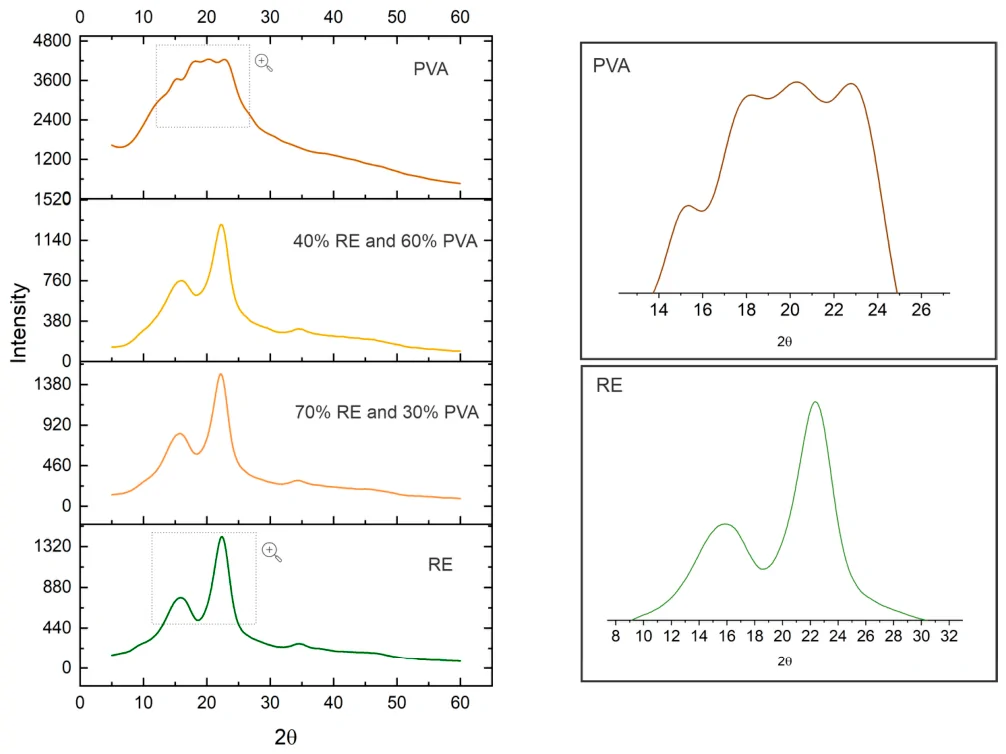
XRD patterns of the RE, 40% RE and 60% PVA, 70% RE and 30% PVA, and PVA samples. Source: Author’s own work.

Figure 4 presents the X-ray diffraction patterns of the wood–plastic composites produced with the highest and lowest concentrations of *Eucalyptus* residue (70% RE and 30% PVA; 40% RE and 60% PVA), as well as those of the individual materials, *Eucalyptus* residue (RE) and PVA. X-ray diffraction was used to evaluate the crystallinity of the composites and to determine whether the materials exhibited predominantly crystalline or amorphous characteristics based on the positions and intensities of the peaks observed in the diffraction patterns.

As expected, the diffraction patterns indicate the presence of characteristic peaks mainly associated with the lignocellulosic phase, particularly cellulose, in both the composite samples and the *Eucalyptus* residue. The peaks at 15.79° and 22.34° are associated with crystalline structures characteristic of cellulose, as reported in the literature [27,28].

PVA exhibits a predominantly amorphous diffraction profile, although it also presents crystalline contributions, as reported in the literature [29]. The peaks observed at 2θ positions of 15.32°, 18.19°, 20.30°, and 22.88° are close to the positions attributed to crystalline structures of PVA, as described by Batista [30].

In the composites, the RE/PVA ratio influences the diffraction patterns. The composite containing 70% RE and 30% PVA exhibits a profile more similar to that of the *Eucalyptus* residue, with a greater contribution from the peaks associated with the lignocellulosic phase. In the composite containing 40% RE and 60% PVA, these peaks exhibit lower relative intensity, while the contributions associated with the polymeric phase become more representative. These differences are mainly related to the relative proportions of the lignocellulosic and polymeric phases present in each composition.

The peak positions near 16° and 22° (2θ) are characteristic of crystalline cellulose structures, associated with cellulose I and the crystallographic planes of this structure. Thus, the XRD results demonstrate the contribution of the lignocellulosic phase to the crystallographic profile of the composites, without interpreting differences in peak intensity alone as evidence of an increase or decrease in the crystallinity index.

### 3.4. Nuclear Magnetic Resonance (NMR)

**Figure 5:**
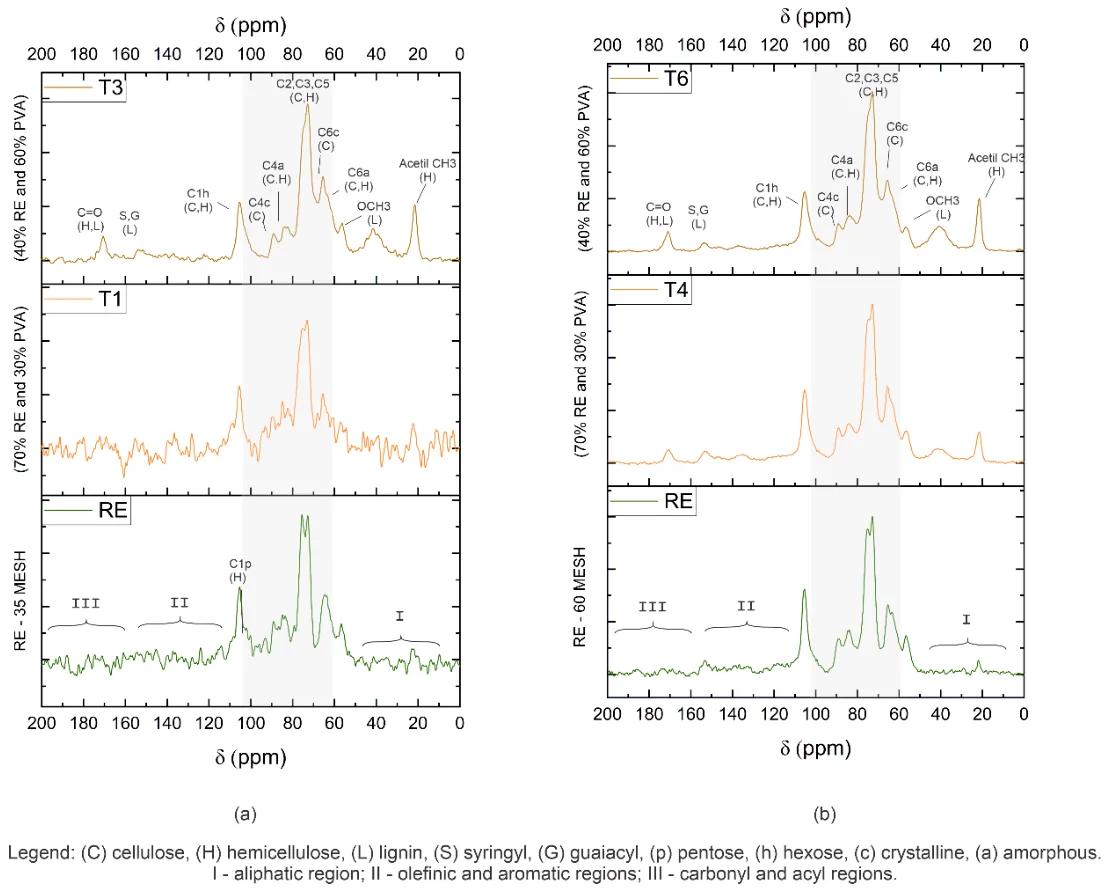
CP/MAS ^13^C NMR spectra obtained for standard samples with particle sizes of (**a**) 35 mesh and (**b**) 60 mesh. Source: Author’s own work.

The CP/MAS ^13^C NMR spectra of the *Eucalyptus* sp. residue samples (RE) of 35 and 60 mesh, as well as those of the treatments with higher residue concentration (T1 and T4—70% RE and 30% PVA) and lower residue concentration (T3 and T6—40% RE and 60% PVA), are shown in Figure 5. The data were defined according to the spectral regions described by Fortunato [12].

The treatments with the highest (T1 and T4) and lowest (T3 and T6) residue concentrations were selected to evaluate the impact of the PVA ratio on the material’s structural organization.

The solid-state ^13^C NMR spectra allowed for the evaluation of structural changes in *Eucalyptus* sp. residues (RE) under the influence of PVA and different particle sizes. Spectral assignments revealed peaks characteristic of cellulose in the 60–110 ppm region, consistent with the literature [31]. The pure ER samples exhibited high structural organization, with well-defined signals at 84–89 ppm (crystalline C4, C4c) and 64–66 ppm (crystalline C6, C6c). Crystallinity was inferred by integrating these signals, following the methodology proposed by Foston et al. [32].

The incorporation of PVA induced significant changes in the NMR profile, depending on the polymer concentration. In the treatments with higher PVA content (T3 and T6, 40% RE/60% PVA), a reduction in the intensity of the C4c and C6c peaks was observed, accompanied by a relative increase in the intensities of the bands associated with the amorphous regions (C4a and C6a). This behavior is attributed to the predominantly amorphous nature of PVA [9], which interferes with the packing of the cellulose chains, leading to an increase in the structural disorder of the composite.

The effect of particle size was also found to be significant in supramolecular organization. Larger particles (RE-35 mesh) exhibited more intense crystalline signals, suggesting more efficient preservation of the ordered regions of cellulose. In contrast, finer particle size (RE-60 mesh) resulted in less pronounced crystalline peaks, a phenomenon possibly associated with greater exposure of reactive surfaces and increased difficulty in interfacial interaction, which, as pointed out by Delviawan et al. [33], tends to compromise the final mechanical performance. It should be noted that, at high PVA contents, these particle size discrepancies become less evident, indicating that the polymer matrix acts as a structural homogenizing agent.

Finally, the preservation of the lignocellulosic components was confirmed by the retention of the characteristic signals of hemicellulose (C1h, C4a) and the aromatic rings of lignin (syringyl and guaiacyl), observed in the range of 50 to 160 ppm. In agreement with Barra et al. [23], the absence of shifts or disappearance of these peaks indicates that the PVA incorporation process does not cause chemical degradation of the eucalyptus constituents, ensuring the integrity of the biomass’s original chemical composition.

### 3.5. Scanning Electron Microscopy (SEM)

**Figure 6:**
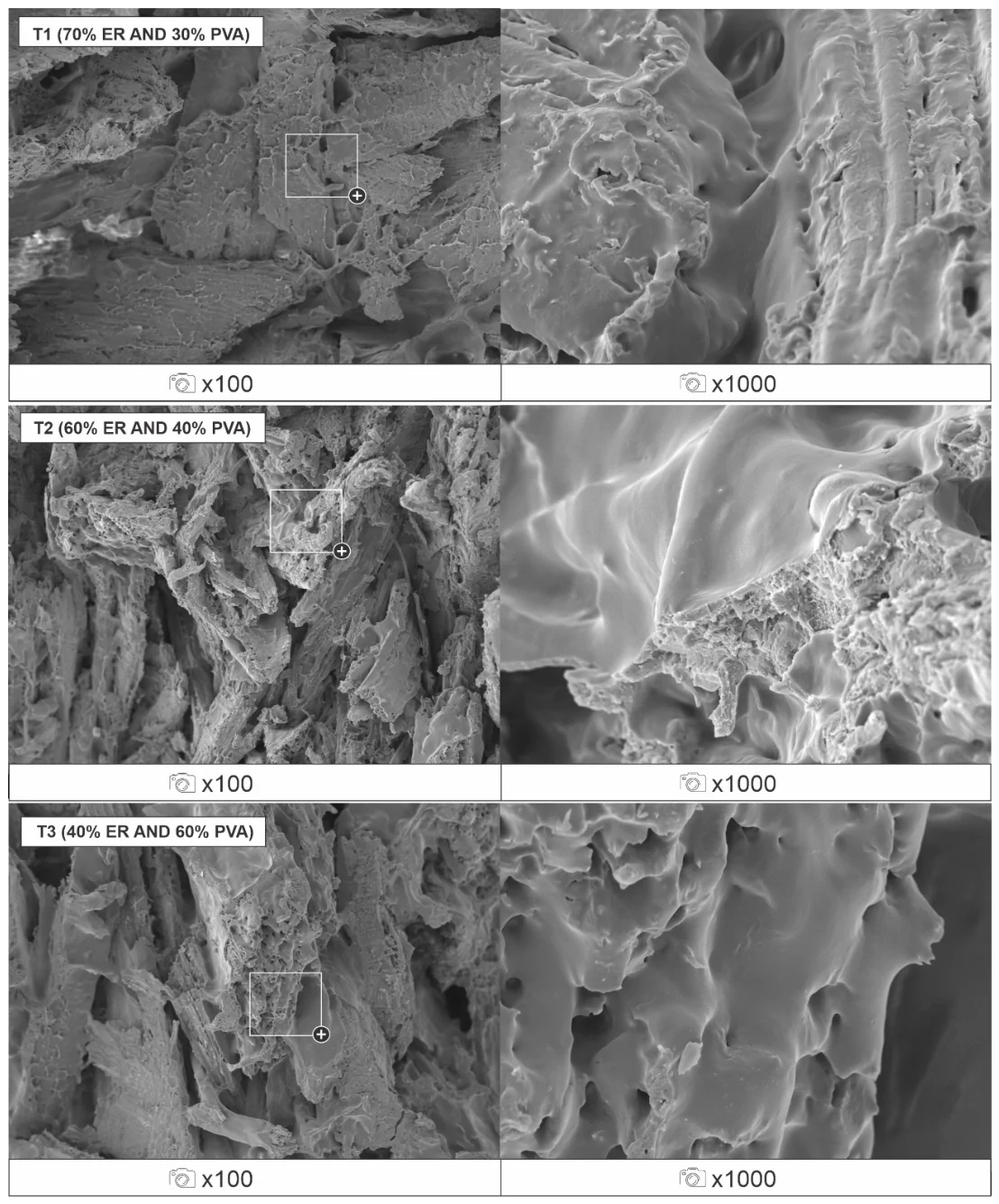
Micrographs of composites T1, T2, and T3 (35 mesh). Source: Author’s own.

**Figure 7:**
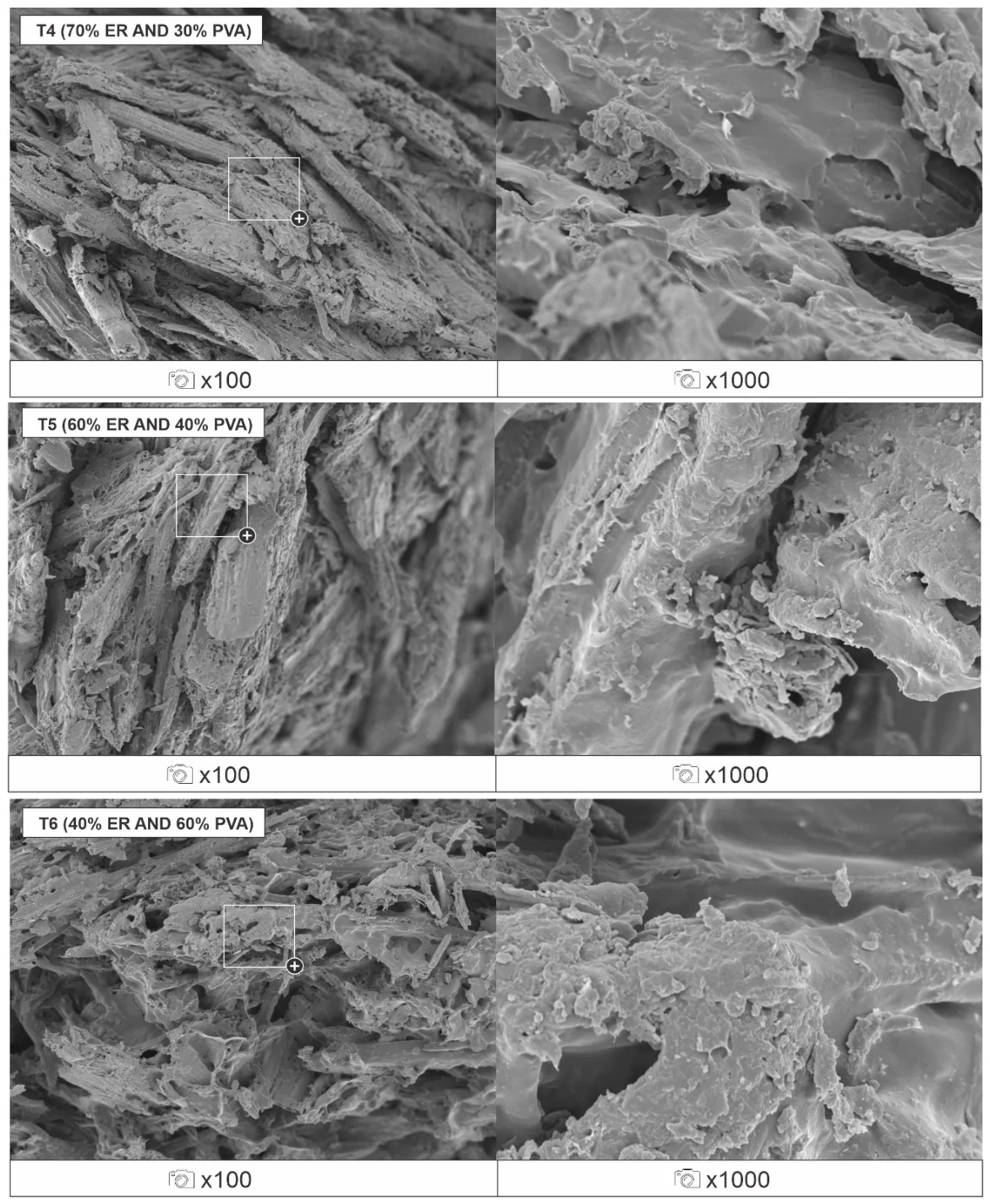
Micrographs of composites T4, T5, and T6 (60 mesh). Source: Author’s own.

Figure 6 and Figure 7 show micrographs obtained from the regions of the tensile test specimens after fracture. In these images, it is possible to identify the interactions between the eucalyptus residues and the polymer matrix at different concentrations and particle sizes. Treatments 1 through 3 are shown in Figure 6, while Treatments 4 through 6 are shown in Figure 7.

The SEM micrographs obtained allowed for the characterization of the fracture morphology and the interface between the *Eucalyptus* sp. residue (RE) and the PVAc matrix, highlighting the influence of polymer concentration and particle size on the distribution of the matrix and the microstructural features of the composites.

In the composites with higher RE content (T1 and T4, 70% RE), the images revealed a heterogeneous fracture surface characterized by interfacial voids, exposed lignocellulosic particles, and pronounced discontinuities between the RE and the polymer matrix. These features indicate regions of limited contact between the two phases and may be associated with incomplete wetting or insufficient matrix coverage of the lignocellulosic surface. As discussed by Hsia [34], differences in the elastic properties of lignocellulosic fillers and polymer matrices can contribute to stress concentration at the interface, potentially influencing the fracture behavior of the composite.

With the increase in PVAc concentration (T3 and T6, 60% PVAc), a notable change in the interface morphology was observed. The micrographs showed a greater extent of polymer coverage over the RE particles, resulting in more continuous polymer-rich regions and fewer visibly exposed lignocellulosic surfaces. However, greater polymer coverage does not, by itself, demonstrate an increase in interfacial adhesion strength. Therefore, the SEM observations indicate changes in the distribution and coverage of the polymer matrix rather than providing direct evidence of stronger interfacial adhesion. The possible interactions between the components were therefore interpreted together with the FTIR and NMR results, which provide complementary information on the chemical and structural characteristics of the composites.

At higher magnification (×1000), differences in the morphology of the voids and fracture features become more evident. The observed voids varied in size and shape, ranging from small and relatively isolated discontinuities to larger and more irregular cavities. Regions showing particle or fiber pullout and interfacial debonding were also observed, indicating that fracture locally occurred through the detachment of lignocellulosic particles from the polymer matrix. These features demonstrate that the fracture process is influenced by the heterogeneous nature of the RE/PVAc interface. Nevertheless, the SEM images provide qualitative morphological information and do not allow the interfacial adhesion strength or the void volume to be quantitatively determined.

When comparing particle sizes, the reduction in particle size (60 mesh) resulted in a more continuous and compact appearance of the polymer-rich regions in some formulations. This behavior may be related to the greater specific surface area of the smaller particles, which requires a larger amount of polymer matrix to achieve adequate surface coverage. However, greater coverage should not be interpreted directly as greater interfacial adhesion, since adhesion strength per unit of interface was not experimentally determined. Thus, the morphology observed by SEM reflects the combined effects of particle size and matrix content on the distribution of the polymer phase and the resulting fracture surface.

The SEM observations are also consistent with the complementary characterization techniques. The FTIR results provide information on the functional groups present in the lignocellulosic residue and PVAc matrix, while the NMR results provide complementary information regarding the structural environment of the composite constituents. The TGA results further demonstrate changes in the thermal behavior associated with the different proportions of RE and PVAc. Therefore, the morphological differences observed by SEM can be interpreted together with the chemical, structural, and thermal results to provide a more comprehensive understanding of the composites. Overall, increasing the PVAc content resulted in greater polymer coverage of the RE particles and changes in the morphology of the fracture surfaces; however, these observations alone do not establish a direct increase in interfacial adhesion strength.

## 4. Conclusions

The results demonstrated the influence of *Eucalyptus* sp. (RE) and PVAc concentrations on the thermal, chemical, structural, and morphological characteristics of the composites. Thermogravimetric analysis showed that the composite containing 70% RE and 30% PVAc exhibited an onset decomposition temperature of 251.63 °C, whereas the composite containing 40% RE and 60% PVAc exhibited an onset temperature of 262.62 °C. The composites presented mass losses of 62.18% and 60.84%, respectively, in the main degradation stage. These results indicate differences in thermal behavior as a function of composition, without supporting the conclusion that increasing PVAc content necessarily reduces thermal stability.

X-ray diffraction patterns showed that the diffraction profiles of the composites were influenced by the relative proportions of the lignocellulosic and polymeric phases, with the composites containing higher RE content showing a greater contribution from the characteristic peaks of crystalline cellulose. ^13^C CP/MAS NMR revealed changes in the structural environment of cellulose as a function of PVAc concentration and particle size. FTIR analysis indicated changes in the intensity of characteristic functional-group bands with the variation in RE/PVAc composition; however, these changes do not provide sufficient evidence to confirm the formation of new covalent bonds or increased hydrogen-bonding interactions.

Morphological characterization by SEM showed greater PVAc coverage of the RE particles at higher polymer concentrations, as well as differences in the fracture morphology associated with particle size and composition. However, the SEM observations provide qualitative morphological information and do not directly demonstrate increased interfacial adhesion strength.

These results contribute to the characterization and valorization of *Eucalyptus* sp. residues through their incorporation into PVAc-based wood–plastic composites. Further studies involving mechanical, physical, and application-specific properties are necessary to assess their suitability for specific applications.

## Figures and Tables

**Table 1:** Distribution of treatments by RE/PVA ratio and particle size.

Ratios	35 MESH	60 MESH
70% RE and 30% PVA	Treatment 1	Treatment 4
60% RE and 40% PVA	Treatment 2	Treatment 5
40% RE and 60% PVA	Treatment 3	Treatment 6

Source: the author.

## Data Availability

The original contributions presented in this study are included in the article. Further inquiries can be directed to the corresponding author.

## Abbreviations

The following abbreviations are used in this manuscript:

ATR	Attenuated Total Reflectance
CP/MAS	Cross Polarization/Magic Angle Spinning
DTG	Derivative Thermogravimetry
FTIR	Fourier Transform Infrared Spectroscopy
NBR	Brazilian Association of Technical Standards (Associação Brasileira de Normas Técnicas)
NMR	Nuclear Magnetic Resonance
PVA	Poly(vinyl acetate)
RE	*Eucalyptus* sp. Residue
SEM	Scanning Electron Microscopy
TG	Thermogravimetry
TGA	Thermogravimetric Analysis
WPC	Wood–Plastic Composite
XRD	X-ray Diffraction
